# Supplementary material for: Machine-learning predicts genomic determinants of meiosis-driven structural variation in a eukaryotic pathogen
Source: Nat Commun. 2021 Jun 10;12:3551. doi: 10.1038/s41467-021-23862-x (PMC8192914; doi:10.1038/s41467-021-23862-x)
Supplement: Supplementary file 1 — Supplementary Information [file 41467_2021_23862_MOESM1_ESM.pdf]

Supplementary Information

Machine-learning predicts genomic determinants of meiosis-driven structural variation in a eukaryotic pathogen

Thomas Badet[1], Simone Fouché[1,2], Fanny E. Hartmann[3], Marcello Zala[2], Daniel Croll[1,*)]

Content

Supplementary Tables (Supplementary Table 1-6)
Supplementary Figures (Supplementary Figure 1-15)

Supplementary Tables

Supplementary Table 1: Phenotypic variance explained by SNPs passing the 5% FDR threshold. Variance is expressed as the correlation coefficient between phenotypic measures and the predicted values from a generalised linear model.

phenotype	variance
ColonyGrowthRate_15C	0.108223423598795
ColonyGrowthRate_22C	0.252298762685275
ColonyGrowthRate_22Cby22C+propiconazole	0.0659651679493618
EC50_Propiconazole	0.0347424481164789
Exp1_Area_mean_14dpi_0.05Propi	0.000721916997095647
Exp1_Area_mean_14dpi_PDA_15C	0.001195367707947
Exp1_Area_mean_14dpi_PDA_22C	0.262875710510477
Exp1_Ratio_14dpi_0.05Propi	0.0284093317682321
Exp1_Ratio_14dpi_15C_22C	0.0254123651960039
MelanizationGreyValue_15C_PDA_11dpi	0.0254234188039681
MelanizationGreyValue_15C_PDA_14dpi	0.00155879608523954
MelanizationGreyValue_15C_PDA_8dpi	0.0558548041141534
MelanizationGreyValue_22C_PDA_11dpi	0.109433052387985
MelanizationGreyValue_22C_PDA_14dpi	0.0816283113579779
MelanizationGreyValue_22C_PDA_18dpi	0.0942562487529362
MelanizationGreyValue_22C_PDA_8dpi	0.0425420320894223
MelanizationGreyValue_22C_PDA+propiconazole_11dpi	0.0383926625084778
MelanizationGreyValue_22C_PDA+propiconazole_14dpi	0.157541274243589
MelanizationGreyValue_22C_PDA+propiconazole_18dpi	0.00903376792834851
PLACP_Toronit	0.137068940434311

Supplementary Table 2: Random Forest model performance metrics on the *Arabidopsis thaliana* dataset for each type of structural variant.

structural_variant	Accuracy	Precision	Recall	F1	Balanced_Accuracy
CP	0.550830397584298	0.713671539122958	0.363716038562664	0.481857764876633	0.583453763962183
DUP	0.768746854554605	0.705731394354149	0.589285714285714	0.642273258077073	0.72782079032079
HDR	0.445143432310015	0.63068526379624	0.39453717754173	0.485414235705951	0.469690113434542
INDEL	0.993205837946653	0.993205837946653	1	0.996591339477339	0.5
INV	0.969300452944137	NA	0	NA	0.5
INVDP	0.757674886763966	0.594042553191489	0.589527027027027	0.591776176345909	0.709279642545771
INVTR	0.471061902365375	0.238042678440029	0.954277286135693	0.381036513545348	0.662969953747458
TDM	0.865123301459487	NA	0	NA	0.5
TRA	0.502516356316054	0.264359071890453	0.941734417344173	0.412830412830413	0.672041497918069

Supplementary Table 3: Confusion matrix of the random forest model applied to the *Arabidopsis thaliana* dataset for each type of structural variant.

structural_variant	number	category
CP	1359	true negative
DUP	2230	true negative
HDR	729	true negative
INDEL	0	true negative
INV	3852	true negative
INVDP	2313	true negative
INVTR	1225	true negative
TDM	3438	true negative
TRA	1302	true negative
CP	1452	false negative
DUP	575	false negative
HDR	1596	false negative
INDEL	0	false negative
INV	122	false negative
INVDP	486	false negative
INVTR	31	false negative
TDM	536	false negative
TRA	43	false negative
CP	333	false positive
DUP	344	false positive
HDR	609	false positive
INDEL	27	false positive
INV	0	false positive
INVDP	477	false positive
INVTR	2071	false positive
TDM	0	false positive
TRA	1934	false positive
CP	830	true positive
DUP	825	true positive
HDR	1040	true positive
INDEL	3947	true positive
INV	0	true positive
INVDP	698	true positive
INVTR	647	true positive
TDM	0	true positive
TRA	695	true positive

Supplementary Table 4: Model performance metrics on the progeny dataset for each type of structural variant.

structural_variant	model	Accuracy	Precision	Recall	F1	Balanced_Accuracy
CP	RF	0.640460691036555	0.0399188092016238	0.776315789473684	0.0759330759330759	0.707070605303458
DUP	RF	0.802203304957436	0.180021953896817	0.792270531400966	0.293381037567084	0.79750838426399
HDR	RF	0.383575363044567	0.0263473053892216	0.741573033707865	0.0508866615265998	0.558494583435239
INDEL	RF	0.0483224837255884	0.0483224837255884	1	0.0921901122522092	0.5
INV	RF	0.998748122183275	NA	0	NA	0.5
INVTR	RF	0.726339509263896	0.0760034158838599	0.89	0.140047206923682	0.806068310220853
INVDP	RF	0.815473209814722	0.227323628219485	0.812	0.355205599300087	0.813852564102564
TRA	RF	0.733350025037556	0.0568888888888889	0.941176470588235	0.107292539815591	0.835463426328249
TDM	RF	0.984977466199299	0.0909090909090909	0.0909090909090909	0.0909090909090909	0.541667622960226
CP	GLM	0.63670505758638	0.038280725319006	0.75	0.0728434504792332	0.69225370086779
DUP	GLM	0.817476214321482	0.18705035971223	0.753623188405797	0.29971181556196	0.787294826312748
HDR	GLM	0.383324987481222	0.0263367916999202	0.741573033707865	0.0508670520231214	0.558366542462127
INDEL	GLM	0.0518277416124186	0.0480382293762575	0.989637305699482	0.0916286879347565	0.496923362136771
INV	GLM	0.990736104156234	0	0	NaN	0.495988969666583
INVTR	GLM	0.762643965948923	0.0810276679841897	0.82	0.147482014388489	0.790585516178737
INVDP	GLM	0.838758137205809	0.242819843342037	0.744	0.366141732283465	0.794542735042735
TRA	GLM	0.77841762643966	0.0631016042780749	0.867647058823529	0.117647058823529	0.822259596655779
TDM	GLM	0.985728592889334	0.0714285714285714	0.0606060606060606	0.0655737704918033	0.527021030807953
CP	ADA	0.635953930896345	0.0400534045393858	0.789473684210526	0.0762388818297332	0.711224846188979
DUP	ADA	0.800200300450676	0.178454842219804	0.792270531400966	0.291296625222025	0.796452139215138
HDR	ADA	0.378818227341012	0.0276461295418641	0.786516853932584	0.0534147272033575	0.578021551166036
INDEL	ADA	0.0483224837255884	0.0483224837255884	1	0.0921901122522092	0.5
INV	ADA	0.998748122183275	NA	0	NA	0.5
INVTR	ADA	0.725087631447171	0.0749574105621806	0.88	0.138147566718995	0.80055469953775
INVDP	ADA	0.814471707561342	0.226309921962096	0.812	0.353966870095902	0.813318376068376
TRA	ADA	0.714822233350025	0.0541215653621982	0.955882352941177	0.102442868400315	0.833264660933146
TDM	ADA	0.984727090635954	0.0625	0.0606060606060606	0.0615384615384615	0.526516107808711
CP	GBM	0.609414121181773	0.0374064837905237	0.789473684210526	0.0714285714285714	0.697697536337014
DUP	GBM	0.790686029043565	0.176772867420349	0.830917874396135	0.291525423728814	0.809702401681828
HDR	GBM	0.3542814221332	0.0269756838905775	0.797752808988764	0.0521866960676222	0.570963472356098
INDEL	GBM	0.0483224837255884	0.0483224837255884	1	0.0921901122522092	0.5
INV	GBM	0.998748122183275	NA	0	NA	0.5
INVTR	GBM	0.72058087130696	0.0738255033557047	0.88	0.136222910216718	0.79824345146379
INVDP	GBM	0.812969454181272	0.226019845644983	0.82	0.354364736387208	0.81625
TRA	GBM	0.724837255883826	0.0559862187769165	0.955882352941177	0.105777054515867	0.838358904437985
TDM	GBM	0.979719579369054	0.0862068965517241	0.151515151515152	0.10989010989011	0.569067346017611

Supplementary Table 5: Correspondance table for the transposable elements annotated by TAIR10 and renamed according to the three-letter nomenclature.

TAIR10_naming	model_naming
DNA	DXX
DNA/En-Spm	DTC
DNA/Harbinger	DTH
DNA/HAT	DTA
DNA/Mariner	DTT
DNA/MuDR	DTM
DNA/Pogo	DTP
DNA/Tc1	DTT
LINE	RIX
LINE/L1	RIL
LTR/Copia	RLC
LTR/Gypsy	RLG
RathE1_cons	RSX
RathE2_cons	RSX
RathE3_cons	RSX
RC/Helitron	DHH
SINE	RSX
Unassigned	XXX

Supplementary Table 6: Deduced positions of the 18 crossover breakpoints identified for chromosome 1. Positions are relative to the respective progeny genome.

chr	estimated_breakpoint_position	progeny
chr_1	119068	A2.2
chr_1	536397	Ztprog01
chr_1	646381	Ztprog11
chr_1	907731	A66.2
chr_1	1849264	Ztprog19
chr_1	2054220	Ztprog45
chr_1	2961623	Ztprog08
chr_1	2975041	A66.2
chr_1	3305410	Ztprog01
chr_1	3497017	Ztprog19
chr_1	3710083	Ztprog11
chr_1	4258413	Ztprog19
chr_1	4296985	Ztprog09
chr_1	4727350	Ztprog11
chr_1	4838961	A2.2
chr_1	4915662	Ztprog01
chr_1	5539986	Ztprog09
chr_1	5779922	Ztprog11

Supplementary Figures

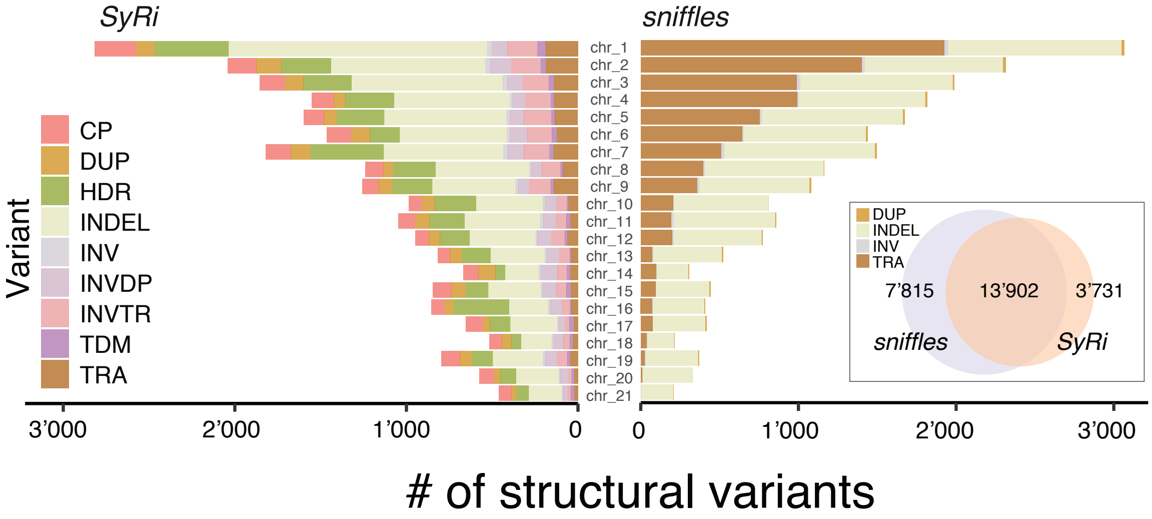

Supplementary Figure 1: Number of structural variants identified on each chromosome using either the whole-genome alignment (SyRi) or the read-mapping method (sniffles) to the IPO323 reference genome. Both methods included a consolidation step for similar variants separated by less than 1,000 bp. Only translocations (TRA), indels (INDEL), inversions (INV) and duplications (DUP) are resolved using the sniffles method, while SyRi enabled the identification of copy variation (CP), highly diverged regions (HDR), inverted duplications (INVDP), inverted translocations (INVTR) and tandem repeats (TDM). The Venn diagram depicts the number of translocations, indels, duplications and inversions that were identified by both methods with overlapping positions.

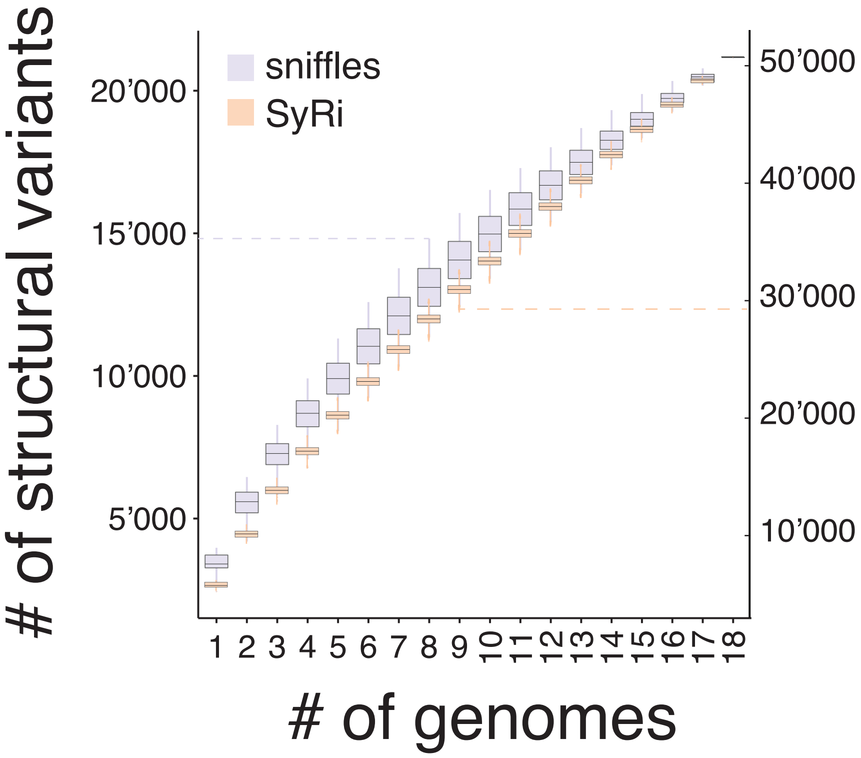

Supplementary Figure 2: Number of structural variants recovered for all permutations over n = 18 genomes. Results are shown for variants identified using either the whole-genome alignment (SyRi; y-axis right side) or the read-mapping method (sniffles; y-axis left side) to the IPO323 reference genome. Boxplots represent the 25th–75th percentile and horizontal lines indicate the median values with whiskers drawn from minimum to maximum values.

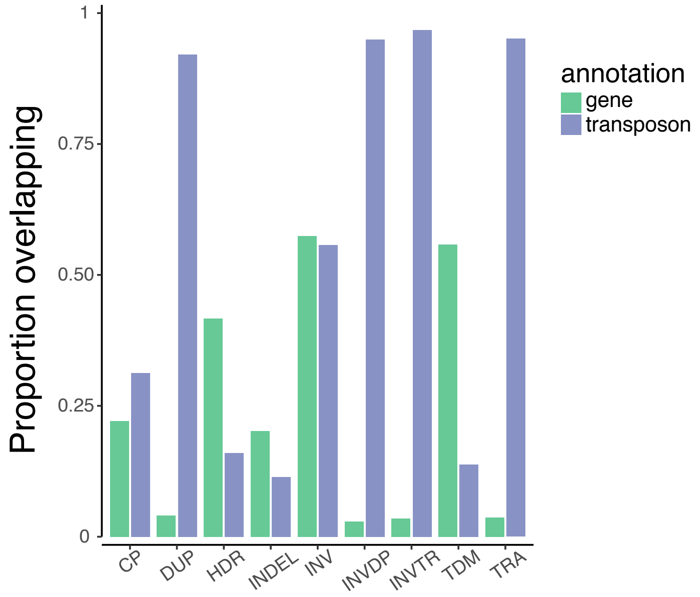

Supplementary Figure 3: Proportion of translocations (TRA), indels (INDEL), inversions (INV), inverted duplications (INVDP), inverted translocations (INVTR), copy variation (CP), highly diverged regions (HDR), tandem repeats and duplications (DUP) overlapping with genes and transposable elements.

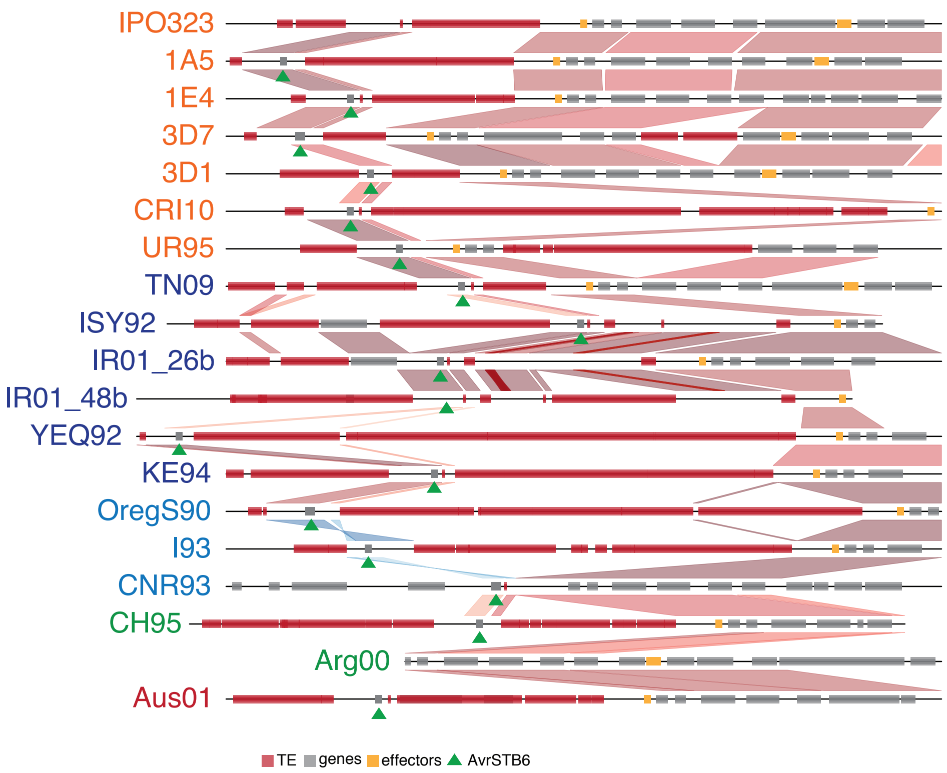

Supplementary Figure 4: Synteny plot of the subtelomeric arm of chromosome 5 where the effector gene *AvrStb6* is located (denoted by the green triangles). The 19 *Zymoseptoria tritici* isolates are coloured as per their location of origin as in Figure 1A. Transposable elements (TE) are shown in red.

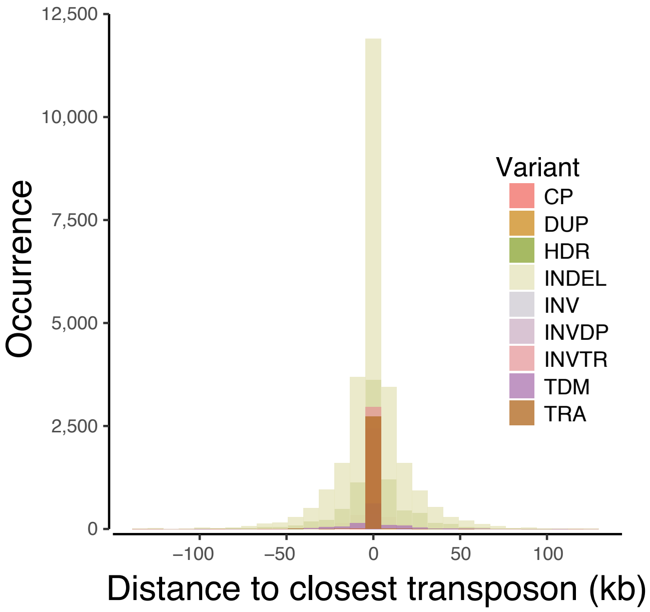

Supplementary Figure 5: Occurrence of translocations (TRA), indels (INDEL), inversions (INV), inverted duplications (INVDP), inverted translocations (INVTR), copy variation (CP), highly diverged regions (HDR), tandem repeats and duplications (DUP) according to the distance to the closest transposable element. The distance is shown in kilobase pair (kb).

SV	model	Accuracy	Precision	Recall	F1
CP	RF	0.63	0.53	0.48	0.50
DUP	RF	0.88	0.67	0.84	0.74
HDR	RF	0.65	0.70	0.75	0.72
INDEL	RF	0.93	0.93	1.00	0.96
INV	RF	0.89	NA	0	NA
INVTR	RF	0.91	0.81	0.90	0.85
INVDP	RF	0.88	0.68	0.85	0.75
TRA	RF	0.91	0.82	0.88	0.85
TDM	RF	0.92	0	0	NaN
CP	GLM	0.61	0.51	0.51	0.51
DUP	GLM	0.88	0.68	0.80	0.74
HDR	GLM	0.65	0.71	0.74	0.72
INDEL	GLM	0.93	0.93	1.00	0.96
INV	GLM	0.89	0.22	0.02	0.04
INVTR	GLM	0.92	0.85	0.86	0.85
INVDP	GLM	0.90	0.72	0.82	0.77
TRA	GLM	0.91	0.88	0.82	0.84
TDM	GLM	0.92	0.14	0.02	0.03
CP	ADA	0.61	0.51	0.47	0.49
DUP	ADA	0.88	0.67	0.83	0.74
HDR	ADA	0.68	0.73	0.76	0.75
INDEL	ADA	0.93	0.93	1.00	0.96
INV	ADA	0.89	NA	0	NA
INVTR	ADA	0.91	0.81	0.90	0.85
INVDP	ADA	0.88	0.67	0.85	0.75
TRA	ADA	0.91	0.81	0.90	0.85
TDM	ADA	0.92	0	0	NaN
CP	GBM	0.61	0.50	0.52	0.51
DUP	GBM	0.87	0.65	0.85	0.74
HDR	GBM	0.68	0.72	0.78	0.75
INDEL	GBM	0.93	0.93	1.00	0.96
INV	GBM	0.89	NA	0	NA
INVTR	GBM	0.91	0.81	0.90	0.85
INVDP	GBM	0.88	0.67	0.86	0.75
TRA	GBM	0.91	0.81	0.89	0.85
TDM	GBM	0.92	0	0	NaN

Supplementary Figure 6: Summary statistics of the models trained to predict translocations (TRA), indels (INDEL), inversions (INV), inverted duplications (INVDP), inverted translocations (INVTR), copy variation (CP), highly diverged regions (HDR), tandem repeats and duplications (DUP) when applied to the pangenome test dataset. For each type of structural variant, four models were trained using random forest (RF), logistic regression (GLM), boosted classification tree (ADA) and stochastic gradient boosting (GBM) algorithms.

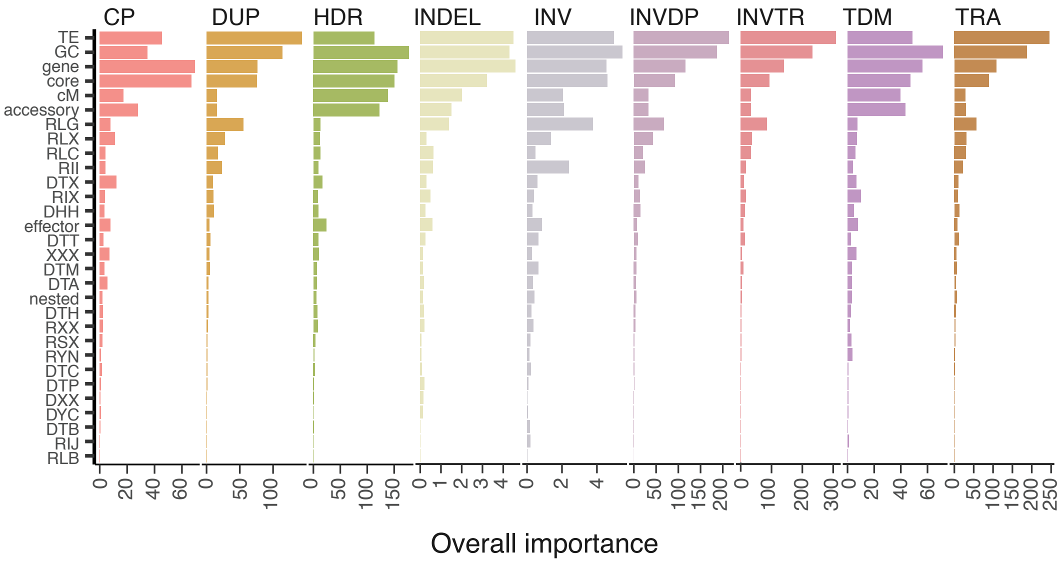

Supplementary Figure 7: Relative importance of the sequence-based metrics used for modelling the occurrence of the nine types of structural variants using the random forest algorithm. Translocations (TRA), indels (INDEL), inversions (INV), inverted duplications (INVDP), inverted translocations (INVTR), copy variation (CP), highly diverged regions (HDR), tandem repeats and duplications (DUP)

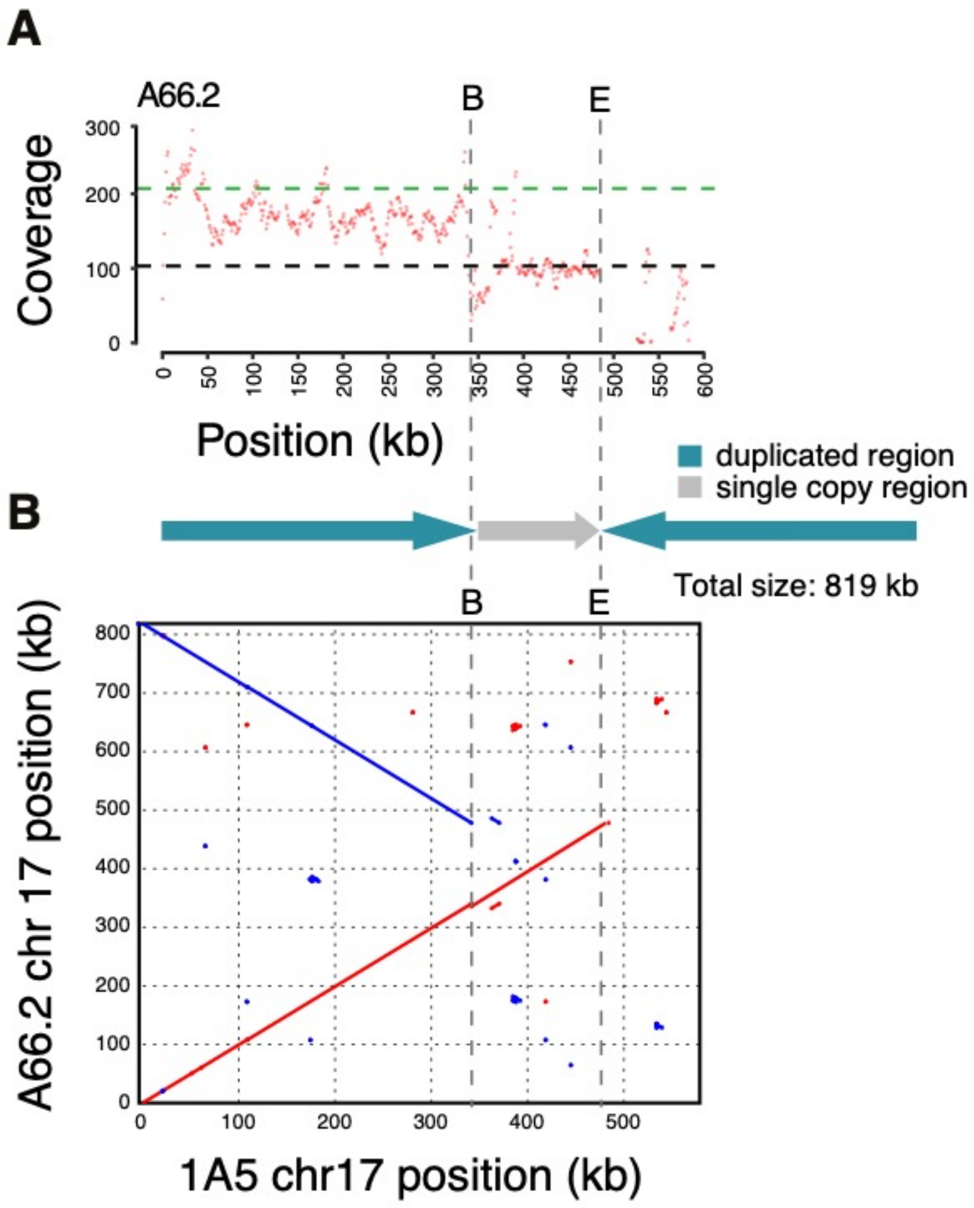

Supplementary Figure 8: A. The coverage and breakpoints of the A66.2 progeny long-reads mapped to the 1A5 parent. Horizontal dashed lines indicate the mean of core chromosome coverage in black and in green the 2-fold mean of core chromosomes coverage. Red dots indicate the mean coverage in 1 kilobase pair (kb) windows (regions with >300X coverage were removed). Vertical dashed lines at B and E indicate positions where split reads map. **B.** Dotplots of the assembled chromosome 17 compared to the 1A5 parental chromosome. Inverted regions are indicated in blue.

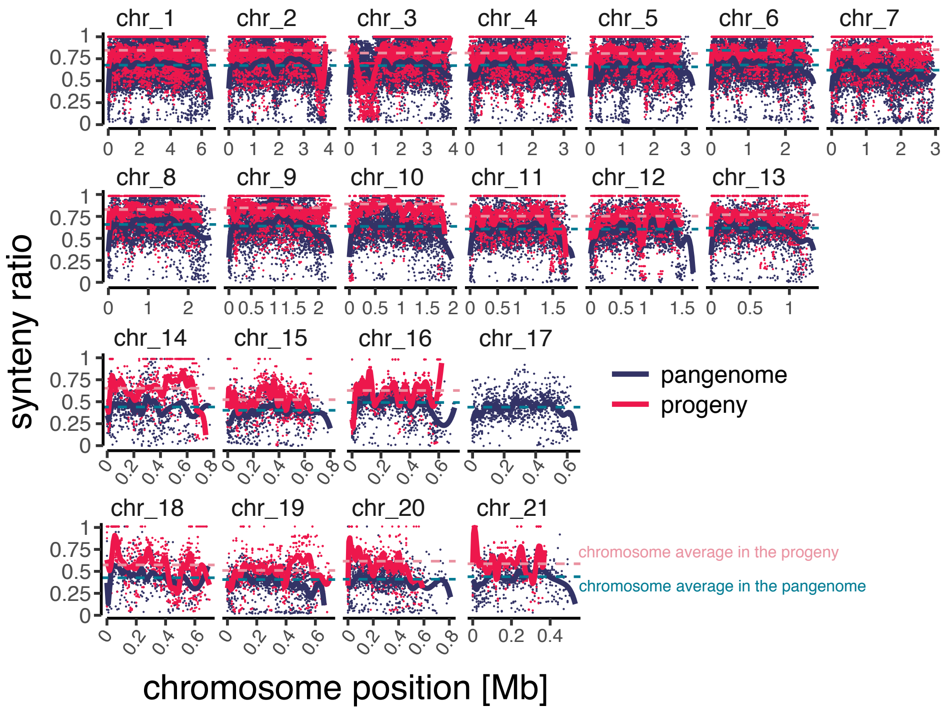

Supplementary Figure 9: Genome-wide synteny expressed as a ratio (see Methods) across non-overlapping 10 kilobase pair (kb) windows in the progeny (in red) and compared to the pangenome (in blue). Coloured curves represent polynomial splines fitting calculated in R using the B-Splines basis with 25 degrees of freedom. Synteny of chromosome 17 is not shown for the progeny as it underwent multiple aberrant non-disjunction events making analyses of collinearity challenging. Chromosome positions are given in megabase pairs (Mb).

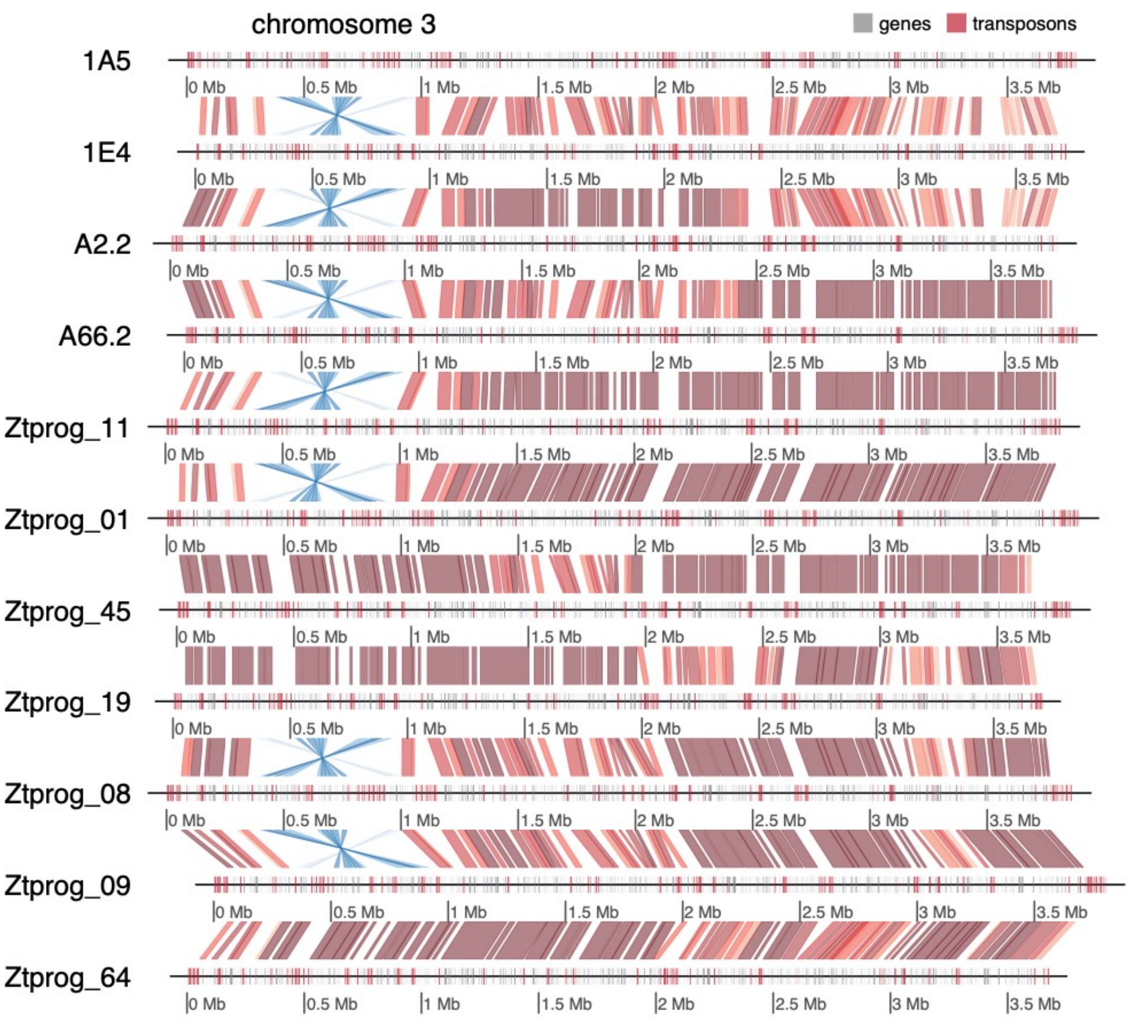

Supplementary Figure 10: Synteny plot of chromosome 3 of different progeny in the pedigree depicting a large segmental inversion in a sub-telomeric region. The region shares low synteny in both the pangenome and the progeny pedigree (see Figure S3).

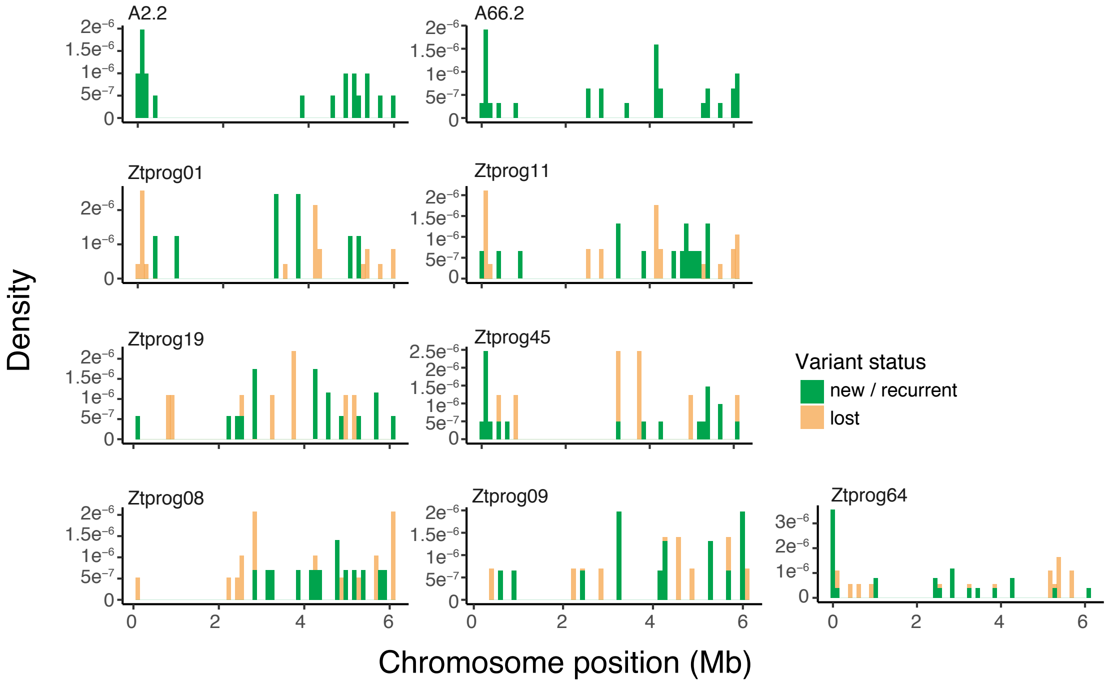

Supplementary Figure 11: Density plot showing the distribution of new and recurrent structural variants compared to lost structural variants on chromosome 1 as observed in the pedigree.

SV	model	Accuracy	Precision	Recall	F1
CP	RF	0.64	0.04	0.78	0.08
DUP	RF	0.80	0.18	0.79	0.29
HDR	RF	0.38	0.03	0.74	0.05
INDEL	RF	0.05	0.05	1.00	0.09
INV	RF	1.00	NA	0.00	NA
INVTR	RF	0.73	0.08	0.89	0.14
INVDP	RF	0.82	0.23	0.81	0.36
TRA	RF	0.73	0.06	0.94	0.11
TDM	RF	0.98	0.09	0.09	0.09
CP	GLM	0.64	0.04	0.75	0.07
DUP	GLM	0.82	0.19	0.75	0.30
HDR	GLM	0.38	0.03	0.74	0.05
INDEL	GLM	0.05	0.05	0.99	0.09
INV	GLM	0.99	0.00	0.00	NaN
INVTR	GLM	0.76	0.08	0.82	0.15
INVDP	GLM	0.84	0.24	0.74	0.37
TRA	GLM	0.78	0.06	0.87	0.12
TDM	GLM	0.99	0.07	0.06	0.07
CP	ADA	0.64	0.04	0.79	0.08
DUP	ADA	0.80	0.18	0.79	0.29
HDR	ADA	0.38	0.03	0.79	0.05
INDEL	ADA	0.05	0.05	1.00	0.09
INV	ADA	1.00	NA	0.00	NA
INVTR	ADA	0.73	0.07	0.88	0.14
INVDP	ADA	0.81	0.23	0.81	0.35
TRA	ADA	0.71	0.05	0.96	0.10
TDM	ADA	0.98	0.06	0.06	0.06
CP	GBM	0.61	0.04	0.79	0.07
DUP	GBM	0.79	0.18	0.83	0.29
HDR	GBM	0.35	0.03	0.80	0.05
INDEL	GBM	0.05	0.05	1.00	0.09
INV	GBM	1.00	NA	0.00	NA
INVTR	GBM	0.72	0.07	0.88	0.14
INVDP	GBM	0.81	0.23	0.82	0.35
TRA	GBM	0.72	0.06	0.96	0.11
TDM	GBM	0.98	0.09	0.15	0.11

Supplementary Figure 12: Summary statistics of the models trained to predict translocations (TRA), indels (INDEL), inversions (INV), inverted duplications (INVDP), inverted translocations (INVTR), copy variation (CP), highly diverged regions (HDR), tandem repeats and duplications (DUP) when applied to the progeny dataset. For each type of structural variant, we show the results of the four models trained using random forest (RF), logistic regression (GLM), boosted classification tree (ADA) and stochastic gradient boosting (GBM) algorithms.

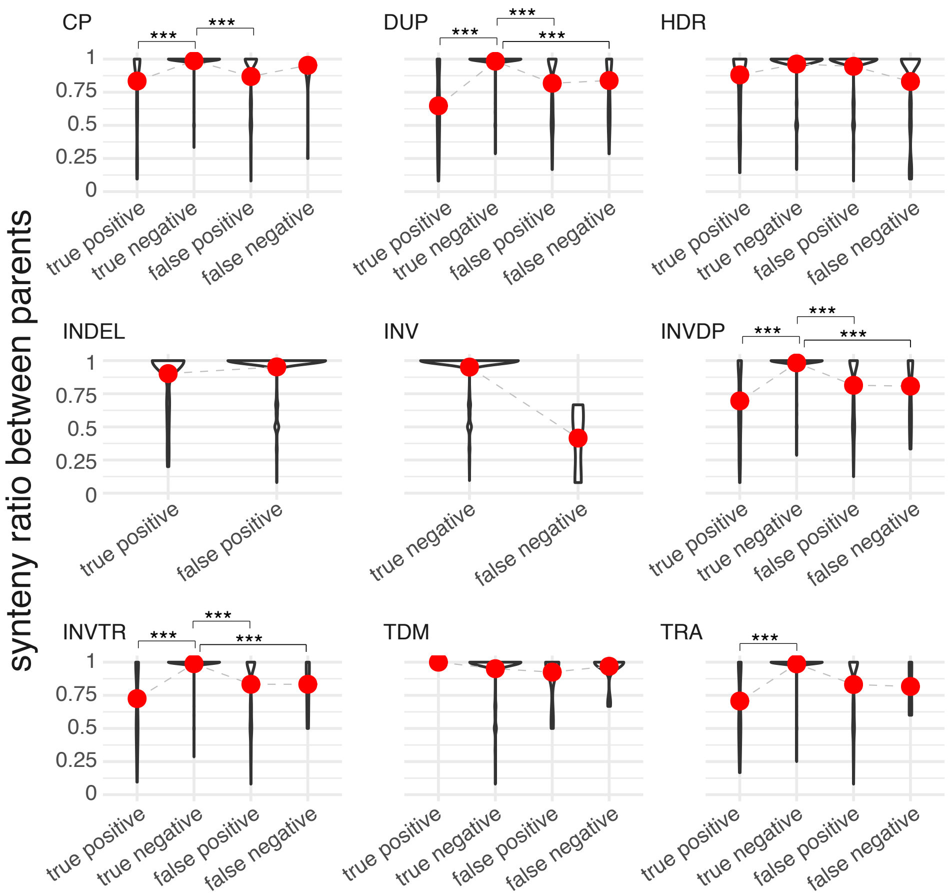

Supplementary Figure 13: Violin plots depicting the distribution of synteny values in 10 kb windows for accurate model predictions (true positives and true negatives) and wrong model prediction (false negatives and false positives). Values for each type of rearrangement predictions are shown separately. Asterisks show significant differences ($p < 1e^{-6}$) based on the Wilcoxon rank-sum test over n = 3,994 windows.

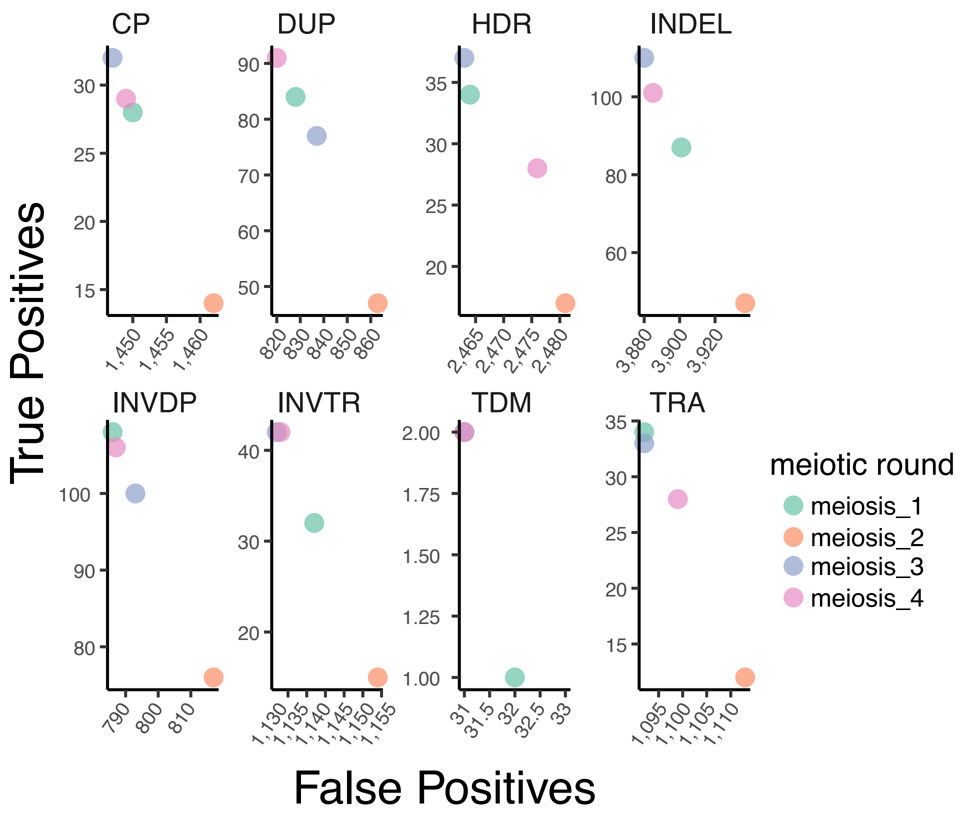

Supplementary Figure 14: Model performance for each progeny set of structural variants generated in the parental round of meiosis (noted M1 to M4, for meiotic round 1 to 4). Performance is shown as the relationship between the true positive rate over the false positive rate. M1 regroups structural variants identified in the progeny A2.2 and A66.2, M2 regroups structural variations identified in progeny Ztprog_01 and Ztprog_11, M3 regroups structural variations identified in progeny Ztprog_19 and Ztprog_45 and M4 regroups structural variations identified in progeny Ztprog_08, Ztprog_09 and Ztprog_64. Results for each type of variant are shown separately.

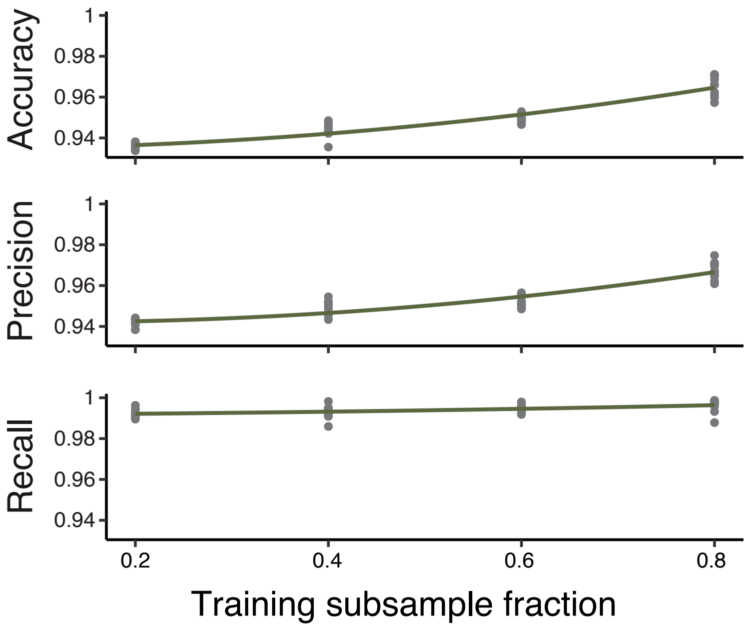

Supplementary Figure 15: Variation of performance metrics for the random forest model trained on 10 random subsamples representing a fraction of 0.2, 0.4, 0.6 and 0.8 of the total pangenome dataset.
