# Supplementary material for: Machine-learning predicts genomic determinants of meiosis-driven structural variation in a eukaryotic pathogen
Source: Nat Commun. 2021 Jun 10;12:3551. doi: 10.1038/s41467-021-23862-x (PMC8192914; doi:10.1038/s41467-021-23862-x)
Supplement: Supplementary file 3 — Reporting Summary [file 41467_2021_23862_MOESM3_ESM.pdf]

nature research

Corresponding author(s): Daniel Croll
Last updated by author(s): 2021/04/27

Reporting Summary

Nature Research wishes to improve the reproducibility of the work that we publish. This form provides structure for consistency and transparency in reporting. For further information on Nature Research policies, see our Editorial Policies and the Editorial Policy Checklist.

Statistics

For all statistical analyses, confirm that the following items are present in the figure legend, table legend, main text, or Methods section.

n/a	Confirmed	
☐	☒	The exact sample size (*n*) for each experimental group/condition, given as a discrete number and unit of measurement
☐	☒	A statement on whether measurements were taken from distinct samples or whether the same sample was measured repeatedly
☐	☒	The statistical test(s) used AND whether they are one- or two-sided<br>*Only common tests should be described solely by name; describe more complex techniques in the Methods section.*
☐	☒	A description of all covariates tested
☐	☒	A description of any assumptions or corrections, such as tests of normality and adjustment for multiple comparisons
☐	☒	A full description of the statistical parameters including central tendency (e.g. means) or other basic estimates (e.g. regression coefficient) AND variation (e.g. standard deviation) or associated estimates of uncertainty (e.g. confidence intervals)
☐	☒	For null hypothesis testing, the test statistic (e.g. *F*, *t*, *r*) with confidence intervals, effect sizes, degrees of freedom and *P* value noted<br>*Give P values as exact values whenever suitable.*
☐	☒	For Bayesian analysis, information on the choice of priors and Markov chain Monte Carlo settings
☐	☒	For hierarchical and complex designs, identification of the appropriate level for tests and full reporting of outcomes
☐	☒	Estimates of effect sizes (e.g. Cohen's *d*, Pearson's *r*), indicating how they were calculated

Our web collection on statistics for biologists contains articles on many of the points above.

Software and code

Policy information about availability of computer code

Data collection	No software was used for data collection
Data analysis	Pangenome assemblies were performed using Canu v1.7.1, Arrow v2.2.2 for polishing and Ragout (Reference-Assisted Genome Ordering UTility) v2.1.1 for chromosome reconstruction. The map presented in Figure 1 was created with rworldmap v_1.3-6 R package. Gene model predictions were performed using BRAKER v2.1 pipeline. Orthogroups were defined using Orthofinder v2.1.2. Transposable elements were de novo annotated using RepeatModeler open-1.0.11 and annotated using RepeatMasker v4.1.0. Progeny genomes were assembled using HGAP version 4 from the SMRTanalysis suite (version 6, release 6.0.0.47841) and Ragout (Reference-Assisted Genome Ordering UTility) version 2.2. Progeny assemblies were polished using Quiver 6.0.0.47835 and visual inspection using IGV (2.4.10). Progeny assembly synteny with the 1A5 parent was assessed using Nucmer (mummer 3.23). Structural rearrangements were mapped using NGMLR software v0.2.7, Sniffles v1.0.10 and SURVIVOR v1.0.7. Whole-genome alignments were performed using nucmer from the MUMmer v4.0.0beta2 suite and synteny inferred with the syri v1.3 software. Fungicide resistance (EC50 values) were calculated using the drc v3.0-1 R package. Phylogenetic relationships were analyzed with the vk suite software (version 0.2.8). Genome-wide association study was performed with TASSEL v.20200220 and GAPIT v3.0 softwares. Sequence-based metrics were recovered with bedtools v2.29.2, blast v2.8.1+ and EMBOSS v6.6.0.0 suites. Machine learning steps were performed under R version 3.6.0 using the packages caret version 6.0-86, ada version 2.0-5, randomForest version 4.6-14 and the models further evaluated using ROCR version 1.0-11 and PRROC version 1.3.1. Figures were plotted using ggplot2 version 3.3.2, GGally version 2.0.0 and genoPlotR version 0.8.9.<br><br>The scripts used to perform the synteny analysis, to map the structural rearrangements and to train the predictive models are available at https://github.com/crolllab/datasets/.

For manuscripts utilizing custom algorithms or software that are central to the research but not yet described in published literature, software must be made available to editors and reviewers. We strongly encourage code deposition in a community repository (e.g. GitHub). See the Nature Research guidelines for submitting code & software for further information.

Data

Policy information about availability of data

All manuscripts must include a data availability statement. This statement should provide the following information, where applicable:

- Accession codes, unique identifiers, or web links for publicly available datasets
- A list of figures that have associated raw data
- A description of any restrictions on data availability

Arabidopsis thaliana datasets were recovered from https://1001genomes.org/data/MPIPZ/MPIPZJiao2020/releases/current/ (orthogroups and structural rearrangements). Transposable elements and gene annotations were retrieved from the TAIR10 database at https://www.arabidopsis.org/download/index-auto.jsp?dir=%2Fdownload_files%2FGenes%2FTAIR10_genome_release and the list of reference NLRs (Col-0) was retrieved from https://ars.els-cdn.com/content/image/1-s2.0-S0092867419308372-mmc3.xlsx (Table S3a, annotated genes under the Accession_Name = Col-0_Ref).
Genome assemblies for the species-wide pangenome are available under the project PRJEB33986. The progeny genomes are available under the project PRJNA645795. Variant calls for GWAS were retrieved from the variant call format (VCF) file deposited in the European Nucleotide Archive (ENA) under the accession numbers PRJEB15502/ERP017268 and the analysis number ERZ330467.

Field-specific reporting

Please select the one below that is the best fit for your research. If you are not sure, read the appropriate sections before making your selection.

☒ Life sciences ☐ Behavioural & social sciences ☐ Ecological, evolutionary & environmental sciences

For a reference copy of the document with all sections, see nature.com/documents/nr-reporting-summary-flat.pdf

Life sciences study design

All studies must disclose on these points even when the disclosure is negative.

Sample size	Pangenome analyses (Z. tritici): n=19 genomes; pangenome analyses (A. thaliana): n=8 genomes; Z. tritici four-generation progeny pedigree, n=9 genomes
Data exclusions	No data was excluded
Replication	Genomes of individuals in populations and a pedigree were analyzed. No experiments were conducted.
Randomization	Individuals and progeny were randomly selected from populations and crosses, but no experiments were performed.
Blinding	Blinding was not performed as no experiments were conducted.

Reporting for specific materials, systems and methods

We require information from authors about some types of materials, experimental systems and methods used in many studies. Here, indicate whether each material, system or method listed is relevant to your study. If you are not sure if a list item applies to your research, read the appropriate section before selecting a response.

Materials & experimental systems

n/a	Involved in the study
☒	☐ Antibodies
☒	☐ Eukaryotic cell lines
☒	☐ Palaeontology and archaeology
☒	☐ Animals and other organisms
☒	☐ Human research participants
☒	☐ Clinical data
☒	☐ Dual use research of concern

Methods

n/a	Involved in the study
☒	☐ ChIP-seq
☒	☐ Flow cytometry
☒	☐ MRI-based neuroimaging

April 2020
