# Supplementary material for: Machine-learning predicts genomic determinants of meiosis-driven structural variation in a eukaryotic pathogen
Source: Nat Commun. 2021 Jun 10;12:3551. doi: 10.1038/s41467-021-23862-x (PMC8192914; doi:10.1038/s41467-021-23862-x)
Supplement: Supplementary file 4 — Description of Additional Supplementary Files [file 41467_2021_23862_MOESM4_ESM.pdf]

Description of Additional Supplementary Files

File Name: Supplementary Data 1
Description: List and positions of all PacBio read-mapping based structural variants identified in the 19-isolate pangenome. Positions are given relative to the IPO323 reference genome

File Name: Supplementary Data 2
Description: List and positions of all whole-genome alignment based structural variants identified in the 19-isolate pangenome. Positions are given relative to the IPO323 reference genome

File Name: Supplementary Data 3
Description: Information on the 24 phenotypic traits measured for genome-wide association studies. Least square mean values based on raw phenotypic data from 106 Zymoseptoria tritici isolates sampled across five populations. MCA = Mean Colony Area ; RCA = Ratio of Colony Area (15°C/22°C) ; * = propiconazole treatment

File Name: Supplementary Data 4
Description: Pairwise Spearman's correlation values for different sequence-based metrics

File Name: Supplementary Data 5
Description: List of different sequence-based metrics used for the correlation study and the subset of 30 metrics used for machine-learning prediction of indels and translocations

File Name: Supplementary Data 6
Description: Model performance metrics of the 20% pangenome subset dataset for each type of structural variant

File Name: Supplementary Data 7
Description: Values of area under the receiver operating characteristic (ROC) curve and precision recall (PROC) for each model tested on the 20% pangenome subset dataset for each type of structural variant

File Name: Supplementary Data 8
Description: Confusion matrix of each model tested on the 20% pangenome subset dataset for each type of structural variant

File Name: Supplementary Data 9
Description: List and positions of all structural variants identified in the 9 progeny. Only newly emerged structural variants are shown (variants absent from any of the two parental genomes). Positions are given relative to the 1A5 reference genome. Names in parentheses are the isolate identifiers in the NCBI database

File Name: Supplementary Data 10
Description: Confusion matrix of each trained model model applied to the progeny dataset for each type of structural variant

File Name: Supplementary Data 11
Description: Table with the 30 sequence metrics used for modelling indels mapped in the Arabidopsis thaliana Col-0 reference genome
